# Comparative efficacy and acceptability of interventions for universal, selective and indicated prevention of eating disorders: study protocol for a systematic review and network meta-analysis

**DOI:** 10.1186/s40337-025-01244-8

**Published:** 2025-04-25

**Authors:** Sandra Schlegl, Felicitas Hirler, Andreas Gerich, Mikkel Højlund, Eric Stice, Tracey Wade, Denise Wilfley, James Downs, Ulrich Voderholzer, Jasmine Perry, Verena Haas, Marco Solmi, Christoph Correll

**Affiliations:** 1 Department of Psychiatry and Psychotherapy, University Hospital, LMU Munich, Nussbaumstrasse 7, 80336 Muenchen, Germany; 2 Department of Regional Health Research, University of Southern Denmark, Aabenraa, Denmark; 3 Department of Psychiatry and Behavioral Sciences, Stanford University, Stanford, CA USA; 4 Institute for Mental Health and Wellbeing, Flinders University, Adelaide, Australia; 5 Department of Psychiatry, Washington University School of Medicine, St. Louis, MO USA; 6 Independent Researcher and Expert by Experience, Cardiff, Wales, UK; 7 Department of Psychiatry and Psychotherapy, University Hospital of Freiburg, Freiburg, Germany; 8 Schoen Clinic Roseneck, Prien am Chiemsee, Germany; 9 Department of Child and Adolescent Psychiatry, corporate member of Freie, Charité Universitätsmedizin Berlin, Universität Berlin and Humboldt-Universität zu Berlin, Berlin, Germany; 10 SCIENCES Lab, Department of Psychiatry, University of Ottawa, Ottawa, ON Canada; 11 Regional Centre for the Treatment of Eating Disorders and On Track, The Champlain First Episode Psychosis Program, Department of Mental Health, The Ottawa Hospital, Ottawa, ON Canada; 12 Clinical Epidemiology Program, Ottawa Hospital Research Institute (OHRI), University of Ottawa, Ottawa, ON Canada; 13 Department of Psychiatry Research, The Zucker Hillside Hospital, Glen Oaks, NY USA; 14 Department of Psychiatry and Molecular Medicine, Donald and Barbara Zucker School of Medicine at Hofstra/Northwell, Hempstead, NY USA

**Keywords:** Anorexia nervosa, Bulimia nervosa, Binge-eating disorder, Eating disorders, Systematic review, Network meta-analysis, Prevention, Universal, Selective, Indicated

## Abstract

**Background:**

Eating disorders (EDs) are severe psychiatric conditions, with prevalence rates ranging from 5.5 to 17.9% in women and 0.6 to 2.4% in men. EDs carry a high risk of chronicity and mortality, highlighting the need for effective prevention strategies. Primary prevention can target the entire population (universal), high-risk groups (selective), or individuals with early signs (indicated). Despite substantial research, prior reviews often show limitations, such as single-author data extraction, lack of quality assessment, reliance on endpoint data, exclusion of obesity prevention programs, or outdated findings. No review has yet evaluated the comparative effectiveness of multiple interventions across risk groups. This article outlines a systematic review and network meta-analysis (NMA) protocol to assess the comparative effectiveness of various ED preventive interventions across different prevention types and populations.

**Methods:**

Eligible studies will include (cluster) randomized controlled trials (RCTs) involving children, adolescents, and adults across a range of settings. Databases to be searched include MEDLINE, Embase, PsycINFO, and CENTRAL. All prevention types (universal, selective, indicated) will be included. Interventions will encompass psychological, educational, physical, and nutritional approaches aimed at preventing EDs, disordered eating, or negative body image and/or reducing risk factors. Coprimary outcomes will be ED diagnostic symptoms, overall ED pathology, ED onset, and intervention all-cause discontinuation (acceptability). A frequentist NMA framework will be used for data synthesis, with sensitivity and subgroup analyses to identify effect modifiers.

**Discussion:**

This first NMA on ED prevention aims to provide valuable insights for clinicians, researchers, policymakers and the public by identifying the most effective interventions and highlighting research gaps. The findings will inform intervention selection for specific populations and guide future prevention strategies to reduce the burden of EDs on affected individuals, their communities, and wider society.

**Clinical trial registration number:**

CRD42024498102.

**Supplementary Information:**

The online version contains supplementary material available at 10.1186/s40337-025-01244-8.

## Background

Eating disorders (EDs) are severe psychiatric illnesses characterized by abnormal eating patterns and weight control behaviors [1]. The prevalence of EDs according to Diagnostic and Statistical Manual of Mental Disorders (DSM-5) criteria ranges from 5.5–17.9% in young women and 0.6–2.4% in young men [2], with the typical onset occurring between the ages of 15 and 23 [3]. Anorexia nervosa (AN) has been reported by 0.8–6.3% of women and 0.1–0.3% of men in their lifetime. Bulimia nervosa (BN) affects 0.8–2.6% of women and 0.1–0.2% of men. Binge-eating disorder (BED) is experienced by 0.6–6.1% of women and 0.3–0.7% of men. Other specified feeding or eating disorders affect 0.6–11.5% of women and 0.2–0.3% of men, while unspecified feeding or eating disorders impact 0.2–4.7% of women and 0–1.6% of men [2]. For those affected, the risk of reduced quality of life and a range of negative health outcomes including mortality is increased, and recovery from EDs often occurs after a chronic and severe course of illness [4]. These factors highlight the importance of prevention efforts.

Primary prevention, as defined by the World Health Organization [5], involves actions that aim to prevent diseases before their onset. It can be categorized into three main types: universal, selective, and indicated prevention. Universal prevention refers to interventions that address an entire population segment without assessing individual risk status. In contrast, selective prevention targets populations with identified heightened risk factors. Lastly, indicated prevention focuses on individuals exhibiting potentially prodromal symptoms of illness, yet falling below the criteria for diagnosis [6].

Presently, there are a considerable number of meta-analyses, systematic reviews, and non-systematic reviews focused on the prevention of EDs. There are broad meta-analytic reviews (e.g. [7], [8], [9]) and focused prevention meta-analyses dedicated to analyzing specific interventions for ED prevention. The latter include reviews on programs such as “Student Bodies”, dissonance-based (DB) interventions, online interventions, and interventions implemented only in school or only in university settings [10–15]. The five most comprehensive meta-analyses and reviews conducted to date are as follows: Le et al. [7] (*N* = 112 studies), Bailey et al. [16] (*N* = 98 studies on prevention), Watson et al. [9] (*N* = 97 studies), Stice, Shaw and Marti [8] (*N* = 68 studies), and Stice et al. [15] (*N* = 56 studies). The only meta-analytic review of trials that tested whether ED prevention programs prevent ED onset was published by Stice, Onipede and Marti [17] and included 15 trials.

Main results of previous systematic reviews and meta-analyses on the prevention of EDs highlight several key findings.

In general, previous studies support the effectiveness of ED prevention programs in addressing various risk factors associated with EDs [7, 8, 9]. These programs also show efficacy in mitigating current or future symptoms of EDs, with effect sizes typically ranging from small to medium [7, 8]. Larger effect sizes are observed in interventions that are selective, interactive, multi-session, tailored for females, targeted at participants > 15 years old, delivered by professional interventionists, and have shorter follow-up periods [8]. Furthermore, programs with body acceptance and DB content, and without psychoeducational content also produced larger effects [8].

Concerning the three types of primary prevention, the bulk of evidence exists for selective prevention, while research on indicated prevention is especially limited [7]. Only two meta-analyses [7, 9] evaluated ED prevention interventions across all three prevention levels.

Among universal prevention strategies, Media Literacy interventions are the best-supported approach [7, 9]. These interventions have shown efficacy in preventing ED risk factors not only in women but also in men [7].

The most supported approaches in selective prevention include DB interventions, Cognitive Behavioral Therapy (CBT), Media Literacy programs, Healthy Weight interventions, and Psychoeducation [7, 9].

Results for indicated prevention approaches are scarcer and vary: CBT interventions are the best-supported approach, demonstrating improvements in ED risk factors and symptoms [9]. However, one review found no effective intervention for reducing ED risk factors [7].

Overall, these findings underscore the importance of tailored prevention strategies, with a focus on specific populations and intervention types, to effectively address the complex nature of evolving EDs. Further research is needed to explore additional effective prevention approaches at all three population levels and to refine existing strategies.

Notably, the most comprehensive reviews available were published more than 6 years ago, indicating a potential gap in recent comprehensive analyses. Furthermore, none of these reviews evaluated how different interventions compare against each other, as they were limited to summarizing direct evidence from individual studies rather than integrating direct and indirect comparisons across multiple interventions.

The previous reviews highlight several areas of opportunity for future systematic reviews and meta-analyses. First, there is a need for methodological improvements, such as utilizing change scores of outcomes and ensuring data extraction is conducted by more than one author [7, 8]. Second, there is a gap in evidence regarding the effectiveness of ED prevention programs in pre-adolescent children and adults, warranting further investigation [7]. Third, future research should not only focus on intervention effects, such as the reduction of risk, but also on true prevention effects, such as preventing the growth of risk, and should include measures of ED onset [9, 17]. Fourth, reviews should explore a broader range of outcomes, including body dissatisfaction, dieting behaviors, thin-ideal internalization, negative affect, eating pathology, bulimic symptoms, body mass index (BMI), drive for thinness, self-esteem [9], and functional outcomes [18]. Lastly, intervention programs promoting physical activity should be examined for their potential role in ED prevention [18]. While psychological and educational approaches dominate ED prevention research, emerging evidence suggests that lifestyle-based interventions, including those promoting physical activity, may also help reduce ED risk and prevent ED onset. However, at the same time abnormally increased physical activity can also be a symptom of an ED [19, 20], or precipitate ED onset in vulnerable individuals, and these approaches have remained underrepresented in prior systematic reviews and meta-analyses. Given their potential for ED prevention, this study will systematically assess the effectiveness of lifestyle-based interventions alongside psychological and educational interventions, providing insights into their comparative impact across different prevention levels.

So far, four network meta-analyses (NMAs) have been conducted on psychological treatments for EDs [21–24]. However, none of the prior NMAs specifically addressed prevention.

An NMA extends traditional meta-analysis by allowing the simultaneous comparison of multiple interventions, even if they have not been directly assessed in head-to-head trials [25, 26]. While traditional meta-analyses synthesize only direct evidence from studies comparing the same two treatments, NMAs incorporate both direct and indirect evidence within a network of studies, increasing the number of quantitative comparisons that are possible. This methodology is especially valuable for comparing multiple treatments that have been studied in different trials, enabling a more comprehensive analysis of their relative effectiveness. Additionally, NMAs facilitate the ranking of interventions, offering clearer guidance on their comparative benefits. Therefore, NMAs play a crucial role in informing policy decisions and establishing guidance for ED prevention implementation.

There is one previously published NMA on the prevention of mental disorders, by Caldwell et al. [27], published in Lancet Psychiatry. The authors presented a systematic review and NMA focusing on interventions aimed at preventing anxiety and depression in children and young people aged 4–18 years, primarily within educational settings. A total of 137 studies, involving 56,620 participants, were included in the analysis. The study evaluated various interventions and their effectiveness based on different outcomes such as self-reported anxiety and depression, wellbeing, suicidal ideation, and self-harm. Key findings included limited evidence supporting the effectiveness of cognitive-behavioral interventions for reducing anxiety, particularly in secondary settings (age 12–18 years). Mindfulness and relaxation-based interventions showed some promise in reducing anxiety symptoms in secondary settings. However, there was insufficient evidence to support any single intervention type for preventing depression. The study highlighted the need for further research, suggesting that current educational setting-based interventions may not be highly effective and proposing exploration of alternative multilevel, systems-based approaches.

## Objectives

The aim of our project is to conduct a systematic review and NMA to evaluate the comparative effectiveness of ED preventive interventions in terms of reducing risk factors, ED symptoms, ED behaviors, ED criteria or ED onset as well as acceptability of interventions. In doing so, this NMA serves as both an update to previous reviews on ED prevention and an extension that allows for indirect comparisons between interventions that were not directly compared in earlier reviews. Moreover, we specifically consider the various levels of primary prevention (universal, selective, indicated) in our analysis, providing a comprehensive assessment of intervention efficacy across different prevention strategies and risk groups. The proposed NMA will answer the following questions:

1. What is the comparative efficacy and all-cause discontinuation (acceptability) of different approaches developed for the prevention of EDs, across different prevention types and populations?
2. Are there important moderators of effect sizes that should guide broad implementation of ED prevention programs?

## Methods

Within this study protocol, we follow the Preferred Reporting Items for Systematic review and Meta-Analysis Protocols (PRISMA-P) 2015 statement [28]. We will follow the PRISMA extension statement for NMAs [29] in our main paper. In accordance with the guidelines, our NMA protocol was registered with the International Prospective Register of Systematic Reviews (PROSPERO, https://www.crd.york.ac.uk/prospero/, registration number CRD42024498102).

### Eligibility criteria

Studies will be selected according to the criteria outlined below.

#### Study designs

We will include randomized controlled trials (RCTs), including cluster RCTs, as they are the gold standard for evaluating treatment effects, minimizing biases related to confounding variables, selection bias, and measurement errors. This allows for stronger causal inferences and more reliable comparisons between interventions. We will exclude controlled, quasi-controlled and non-randomized clinical trials or cluster trials, before-after studies, prospective and retrospective comparative cohort studies, cross-sectional studies, case series, and case reports. We will include only main efficacy papers of these RCTs and exclude study protocols of RCTs, secondary analyses of RCTs (unless further outcomes or data on further follow-up timepoints reported), re-analyses of RCTs, qualitative papers related to the RCTs and cost-effectiveness studies.

#### Participants

We will include studies examining children (6–12 years), adolescents (13–17 years), and adults (18–65 years), across the age range. For the universal prevention review, RCTs focusing on entire populations or subpopulations of the general population, such as classes or schools, will be considered. For the selective and indicated prevention review, studies including at-risk or high-risk individuals will be included. Examples of at-risk populations (selective prevention) are: established risk factors, such as biological female, high weight and shape concerns and/or high drive for thinness, persons with type 1 diabetes and athletes. Examples of at high-risk populations (indicated prevention) are: subthreshold ED symptoms, recurrent binge eating, and/or compensatory behaviors at a frequency of less than once a week, some transient or mild symptomatic behaviors. We will exclude samples with persons with diagnosed EDs.

#### Interventions

We will include universal, selective, and indicated prevention interventions. As interventions we will consider psychological, psychosocial, educational, physical and nutritional interventions with the aim to prevent negative body image, disordered eating or EDs and/or to prevent/reduce risk factors. Examples of prevention approaches that will be considered are: Cognitive Dissonance, CBT, Psychoeducation, Media Literacy, Therapeutic Writing, Obesity Prevention (also targeting ED prevention), Healthy Weight, Self-esteem Enhancement, Physical Activity, Interpersonal Psychotherapy, Perfectionism, Lifestyle Modification, Evaluative Conditioning, Mindfulness, Yoga, Dietary Intervention, Self-compassion, Multicomponent, and “One-shot” (one session intervention). We will include interventions in any format (e.g., individual, dyad, family, class/group or school level). We will include all modes of delivery (e.g., by clinicians, trained facilitators, teachers, coaches, undergraduates, peer leaders, counselors, self-help). Interventions may be delivered either face-to-face (f2f) or by an electronic device (computer, smartphone, app, wearable). We will exclude treatments for persons with a diagnosis of ED.

Additionally, we will exclude interventions that address body image concerns in response to medical, reproductive, or life-stage changes (e.g., pregnancy, postpartum, postmenopause, mastectomy, infertility), as these are distinct from interventions designed to prevent EDs or disordered eating. The body image concerns in these groups often arise due to specific physiological, hormonal, or medical factors rather than the sociocultural and psychological mechanisms that contribute to ED risk in other at-risk populations. Moreover, including these studies would introduce conceptual as well as population and intervention heterogeneity in the NMA, violating transitivity assumptions and making it difficult to validly assess the comparative effectiveness of interventions targeting ED prevention.

#### Comparators

We will consider all types of control groups for the analysis, categorized into at least five broad groups: no treatment/assessment only, class/treatment as usual, minimal intervention, nonspecific intervention, and waitlist-control. If an RCT used psychoeducation as the control condition, we will classify it as a minimal intervention. Since this is an NMA, and head-to-head comparisons will enhance the accuracy of the network, we will also include RCTs that designate active interventions as control arms. In such cases, if an active intervention aligns with one of the interventions outlined in the intervention section, we will treat it as an intervention arm in our analysis.

#### Outcomes

##### Coprimary outcomes

1. ED symptoms/diagnostic symptoms (e.g., Eating Disorder Diagnostic Interview, EDDI; Eating Disorder Diagnostic Scale, EDDS; Eating Disorder Examination Interview, EDE – Diagnostic items; Eating Disorder Examination Questionnaire, EDE-Q – Diagnostic items).
2. Overall eating pathology will be assessed using global scores from measures such as the EDE/EDE-Q, Eating Attitudes Test (EAT), or Eating Disorder Inventory (EDI). Alternatively, when a global ED psychopathology measure is not available, an average score of at least three individual ED-core domains (e.g., body dissatisfaction, thin ideal internalization, weight concerns, shape concerns, drive for thinness, eating concern, dieting) will be used. If fewer than three domains are reported, these will be treated as secondary outcomes and not combined into the primary outcome.
3. ED development/ED onset (e.g., Clinical interview based on DSM or International Statistical Classification of Diseases and Related Health Problems (ICD) criteria).
4. Study-defined intervention all-cause discontinuation (acceptability).

##### Key secondary outcomes

1. Specific ED cognitions (all being risk factors for EDs).
  - Body dissatisfaction.
  - Thin ideal internalization.
  - Weight concern.
  - Shape concern.
  - Eating concern.
  - Drive for thinness.
  - Dieting (Dieting is classified as a cognitive outcome rather than a behavioral outcome because measures of dieting and dietary restraint do not correlate well with objective measures of dietary intake. These measures primarily reflect cognitive aspects of dietary restraint, such as perceived food restriction and weight control attitudes intentions, rather than actual dietary behaviors).
2. Specific ED behaviors (restrictive eating, binge-eating, purging, excessive exercise, etc.).
3. BMI, %mBMI (percentage of median BMI), BMI percentile etc.
4. Positive outcomes:

- Body appreciation, Body acceptance, Body satisfaction.

##### Additional outcomes

1. Media internalization.

##### Related non-ED outcomes

1. Composite of negative affect, depression, anxiety.
2. Negative affect.
3. Depression.
4. Anxiety.
5. Self-esteem.
6. Self-compassion.

We will include only RCTs with at least one relevant outcome (Coprimary or Key secondary). We will extract outcomes in all data forms (e.g. dichotomous, continuous) as reported in the included studies.

In Supplemental Table 1, we present a preliminary codebook for the categorization of questionnaires by outcomes. It should be noted that this list is not exhaustive.

#### Timing

We will consider RCTs with a posttest or pretest and posttest assessment. The primary outcome timepoint will be end of treatment (EOT). We will also consider results for the following follow-up intervals after cessation of the intervention in terms of maintenance effect: 1–3 months, 4–6 months, 7–12 months, 13–24 months, 25 + months.

#### Setting

There will be no restrictions in terms of setting.

### Information sources

We will search the following databases for intervention RCTs: MEDLINE, Embase, PsycINFO, CENTRAL (Cochrane Central Register of Controlled trials). We will include all peer reviewed RCTs, without a publication date or language restriction. Before the final analysis, we will repeat the search to check for the latest publications.

Furthermore, we will review the reference lists of included studies and previous systematic reviews or meta-analyses. We will include dissertations identified by the regular search and unpublished studies from ClinicalTrials.gov and the EU Clinical Trials Register to minimize publication bias while ensuring methodological rigor. Excluding other types of unpublished studies, such as conference abstracts and internal research, maintains data quality. If a dissertation identified by the regular search is later published as a peer-reviewed journal article, we will include the published version instead. For studies from ClinicalTrials.gov and the EU Clinical Trials Register, only unpublished data will be considered, as published results from these registries are already included. If data from unpublished studies on ClinicalTrials.gov or the EU Clinical Trials Register is incomplete, we will attempt to contact the study authors to request additional details. If no response is received within a predefined time frame, we will use the available data and assess the study’s risk of bias accordingly. To assess the impact of including unpublished studies, we will conduct a sensitivity analysis excluding all data from sources that have not been published in a peer-reviewed journal, including dissertations and other unpublished studies.

### Search strategy

The specific search strategy was created by a librarian with expertise in systematic review searching. The draft MEDLINE search strategy is included in Supplemental Material 2.

### Data management

Literature search results will be uploaded to Covidence Software [30], an internet-based software program for managing and streamlining systematic reviews and facilitating collaboration among reviewers during the study selection process. Duplicates will be excluded. Covidence will also provide a PRISMA flow diagram once the screening process is completed.

### Selection process

Two researchers will independently assess the titles and abstracts generated by the search to determine their alignment with the inclusion and exclusion criteria. Full reports will be acquired for all titles that seem to fulfill the inclusion criteria or in cases of uncertainty. Author pairs will then evaluate the full-text reports to ascertain their compliance with the inclusion criteria. If necessary, additional information will be requested from the authors of the studies to address any eligibility concerns. Disagreements will be resolved through discussion with a third reviewer or seeking further information from the study authors. The reason for excluding trials will be documented in Covidence.

### Data collection process

We will design and use a structured data extraction form including demographic information, methodology, intervention details, and all reported relevant outcomes of included RCTs. Extracted data will include the following:

- Study characteristics: first author, last author, publication year, country/continent, journal, number of arms, setting (school, college, community, university), mode of delivery (clinician-led vs. other), mode of delivery (f2f vs. digital), participant level (group vs. individual), type of RCT (parallel, cluster, pragmatic), sample size, method of analysis (intention-to-treat vs. completer), timeframe of follow-ups (on treatment and, possibly, off treatment), blinding of raters, investigator’s allegiance (i.e., whether the study authors are associated with or have a theoretical preference for a particular intervention being tested).
- Participant characteristics: sample size, sex, gender, age, ethnicity, socio-economic indicators, risk factors such as high weight and shape concerns and drive for thinness, persons with type 1 diabetes, athletes.
- Intervention and comparison group details: type of approach, specific intervention components, if available specific name of the program, manualization of the intervention, duration of the intervention, number of sessions, intensity of interventions, targeted ED type (i.e., whether the intervention is designed to prevent a specific ED, such as AN, BN, BED, as opposed to aiming to reduce any ED risk without focusing on a particular diagnosis).
- Outcome measures and data: standardized interviews or questionnaires, BMI, ED onset, acceptability, losses to follow-up and reasons.

Two researchers will extract the data independently in an excel spreadsheet. Additionally, the two reviewers will classify independently the interventions into the three prevention types following Gordon’s classification of prevention, modified by the US Institute of Medicine [6]. They will also assign approaches to the nodes of the NMA.

Disagreements will be resolved through discussion with a third reviewer or seeking further information from the study authors. Decisions will be recorded in an extra column within the master excel file. In case of missing data, we will try to contact the study authors for unreported data or additional details.

### Risk of bias in individual studies

We will evaluate the risk of bias according to the revised Cochrane risk-of-bias tool for randomized trials (RoB2) [31]. We will judge allocation sequence generation, concealment of allocation, blinding of research personnel and participants, outcome assessor blinding, selective reporting of outcomes, attrition, bias by sponsorship, outcome data completeness and other sources. Two researchers will assess the risk of bias of the included studies independently. Disagreements will be resolved through discussion with a third reviewer or seeking further information from the study authors.

### Data synthesis

#### Measures of treatment effect

##### for continuous outcomes

Continuous outcomes will be analyzed using standardized mean differences (95% confidence interval, CI) because we expect different measurement scales for the specific outcomes. If more than one questionnaire is used for the same outcome, we will extract data from all relevant questionnaires and aggregate these prior to meta-analysis to account for the variation between different measures.

For effect size calculations, we will prioritize the outcome metric that predominates in the data to minimize heterogeneity in the network. Based on typical reporting patterns, this is expected to be endpoint scores. However, if baseline values differ by *≥* 10% and the standard deviation (SD) of the change score is not *≥* 3x of the mean change score, we will use the change score instead. Sensitivity analyses will be conducted using the opposite approach, prioritizing change scores wherever possible, including using calculated change scores to assess robustness of the results. This approach will allow us to assess the consistency of our findings under different methodological and bias assumptions, including the potential impact of selective reporting in the published literature [32].

To assess baseline heterogeneity across included studies, we will evaluate differences in baseline severity values. A study will be classified as an “outlier” if its baseline severity values deviate by more than 0.25 SD from the pooled mean of all included studies, as suggested by Stuart et al. [33]. This classification will be used in sensitivity analyses to determine whether the inclusion of such studies significantly influences the main findings.

The type of prevention will inherently reflect the baseline severity of symptoms, with individuals receiving indicated prevention having higher symptoms, those with selected prevention having more risk factors, and the universal prevention RCTs enrolling the general population, where questionnaire values are expected to match the age/sex-corresponding normative values at baseline. To the best of our knowledge, there is no consensus on how to classify severity of symptoms across frequently used tools, nor is there a consensus on how to convert scores across tools. This lack of standardization poses feasibility and validity challenges for conducting sensitivity analyses based on baseline values. Therefore, we have decided not to conduct such analyses in this study, at least not for studies investigating universal prevention or selective prevention. However, for indicated prevention, where by definition, attenuated symptomatology is present, we propose to attempt a sensitivity analysis across two types of study populations: (i) studies where the baseline score is within 1 SD of the mean score on a given symptom severity scale in patients with manifest ED, and (ii) studies where the baseline score is > 1 SD below the mean score on a given symptom severity scale in patients with manifest ED.

##### for dichotomous outcomes

Dichotomous data (e.g., ED onset) will be analyzed by calculating the risk ratio (RR) along with a 95% CI. It has been demonstrated that RR is more easily understood compared to the odds ratio (OR), and clinicians often interpret OR as RR, resulting in an overestimation of the effect. If results are presented as OR and hazard ratio (HR), these will be converted to RR whenever possible by using the MetaConvert R package [34]. Additionally, for dichotomous outcomes, we will calculate the number-needed-to-treat (NNT) to improve clinical interpretability by dividing 1 by the absolute risk difference in each categorical outcome in case that the group comparison is statistically significant for that outcome.

### Data analysis

Separate NMAs will be conducted for the three types of prevention. In addition, separate NMAs will be conducted for each outcome to account for potential differences in intervention efficacy across ED-related symptoms and behaviors. This approach will provide more detailed insights into intervention effectiveness across specific outcome domains that can further guide clinical care. We will estimate standardized mean differences and RRs with 95% CIs using random effects NMA in a frequentist framework by using R and the netmeta package [35].

Global [36] and local [37] inconsistencies for the NMA will be measured, and the CINeMA framework [38] will be used to assess the confidence in evidence for outcomes. To visualize the available evidence, network and forest plots of change in primary and secondary outcome measures and dropouts at EOT will be presented. In the network plots of treatment comparisons, the size of every node will be proportional to the number of randomized participants. The width of the lines will be proportional to the number of trials comparing two treatments and the color of each edge will represent risk of bias. For each outcome at each timepoint, we will calculate a hierarchy of the competing interventions on the basis of a Surface Under the Cumulative Ranking curve (SUCRA) and mean ranks [39]. SUCRA values will be expressed as percentage, showing the relative probability of an intervention to be among the best options. Publication bias will be measured by visual inspection of funnel plots and Egger’s tests [40].

#### Planned approach for NMA feasibility and alternative analyses

We hypothesize that a common comparator will be available across studies, ensuring a connected network for an NMA. In case a common comparator will only connect some, but not all interventions, disconnected networks will be analyzed separately. However, if key assumptions of an NMA are not met, such as transitivity, homogeneity, or consistency, alternative approaches will be considered as detailed below.

To assess the feasibility of an NMA, we will first evaluate network connectivity and assess transitivity by comparing key study and population characteristics across interventions. Consistency will be examined using both statistical methods (e.g., node-splitting and design-by-treatment interaction models) and visual inspection of network plots. If substantial inconsistency or heterogeneity is detected, we will proceed with alternative analyses.

This means that when an NMA is not appropriate for specific comparisons or subgroups, we will conduct pairwise meta-analyses for direct comparisons where sufficient data are available. If neither NMA nor pairwise meta-analysis is feasible due to limited data, we will provide a structured narrative synthesis summarizing available evidence. Any limitations affecting the feasibility of an NMA will be reported transparently, along with a discussion of their implications for interpretation.

### Dealing with missing outcome data and missing statistics

We will use published SDs whenever available. In cases where standard errors (SEs) are provided instead of SDs, we will convert them to SDs [41]. If neither SDs nor SEs are available, we will first attempt to contact the original authors for the necessary information. If no data can be obtained, we will then proceed with imputation methods. For change score outcomes, if no SDs can be obtained, we will estimate them using a pre/post correlation coefficient of 0.5, as described in Sect. 6.5.2.8 of the Cochrane Handbook for Systematic Reviews [42]. Sensitivity analyses will be conducted using alternative assumed correlation coefficients (e.g., 0.3 and 0.7) to further assess the robustness of the results. For endpoint outcomes, SDs will be used as reported in the studies or imputed using established methods, including estimating SDs from *p*-values, CIs, or t/z-values, following the guidelines specified in Sect. 6.5.2 of the Cochrane Handbook for Systematic Reviews [42].

### Additional analyses (investigation of heterogeneity)

We will perform subgroup analyses investigating potential factors that could modify the coprimary outcomes:

1. Setting: school vs. university level.
2. Participant level: individual vs. group.
3. Mode of guidance: clinician-led vs. other.
4. Mode of delivery: f2f vs. electronic device.
5. Number of sessions: 1, 2–4, 5–8, >8.
6. Intensity of intervention: once, weekly, biweekly, monthly.
7. Targeted ED type: AN, BN, BED, or any ED.
8. Age group: mean age < 18 vs. 18 or greater.
9. Individual at-risk groups: e.g. female, high weight and shape concerns and drive for thinness, persons with type 1 diabetes, athletes.

Furthermore, we will perform sensitivity analyses on the coprimary outcomes as follows:

1. Compare the results of studies that presented only completer analyses vs. those that used an intent-to-treat approach.
2. Compare the results of studies with high risk of bias in the overall domain vs. those without high risk of bias.
3. Compare the results in studies by RCT design (parallel, cluster, pragmatic).
4. Compare the results in studies where raters were masked vs. not.
5. Compare the results in studies where the investigator had allegiance to the tested intervention vs. not.
6. Compare the results using the predominant outcome metric (either endpoint or change scores) vs. the other outcomes metric (either change or endpoint scores).
7. Compare the results including and excluding outliers based on baseline heterogeneity.
8. Compare the results of studies with baseline severity within 1 SD of the mean vs. those with baseline severity > 1 SD below the mean on a given symptom severity scale in indicated prevention.
9. Compare the results of studies that were published in peer-reviewed journals vs. those that were not.

### Involvement of stakeholders with lived experience and at-risk status

Stakeholder (e.g., client, clinician) involvement is appropriately becoming an increasingly important aspect of clinical research. This welcome development is evidenced by initiatives, such as INVOLVE (UK) [43], which provides comprehensive guidelines on public involvement in research, including mental health; PCORI (Patient-Centered Outcomes Research Institute) (USA) [44], which emphasizes patient-centered outcomes research and the involvement of patients throughout the research process; SPOR (Strategy for Patient-Oriented Research) (Canada) [45], a framework for integrating patient perspectives into research; and the toolkit for consumer and community involvement in Health and Medical Research (Australia) [46]. However, despite the growing inclusion of people with lived experience (PWLE) of an ED and their carers or support network perspectives in ED research, these viewpoints have not been extensively or systematically integrated into clinical trials [47] and have to the best of our knowledge never been included in an NMA, meta-analysis, or systematic review in the field of EDs. PWLE and at-risk status might be especially critical stakeholders in prevention research. Their knowledge and expertise, derived from firsthand experiences, will enrich the research and ensure that prevention strategies are relevant, practical, and attuned to the real-world needs and concerns of those most affected or at risk.

For the development of the study protocol, we initially invited two PWLE to participate as team members and co-authors. One person is an expert by experience (having had multiple ED diagnoses over time) from the UK, who also represents a diversity perspective (male, LGBTQ+ community). The other person is a research coordinator in the field of EDs who has personal experience with an ED and has been trained to implement and deliver an ED prevention program, which she has successfully conducted multiple times in a college setting.

For the actual study and the main paper, we will expand participation by including two additional individuals: one currently receiving treatment for an ED, and another who has recently recovered (within the last two years). To ensure that the selected individuals can provide constructive and relevant feedback, we will include individuals who are (i) either currently in treatment or have recently recovered within the last two years, (ii) are stable enough to participate in research discussions as assessed by their clinician if needed, (iii) are able to provide meaningful feedback on research findings, and (iv) willing to contribute to relevant aspects of the study, but without being required to engage in all aspects of the study conduct. Additionally, we plan to include one person with a former at-risk status who received a prevention intervention and did not develop an ED. This broader representation aims at ensuring that perspectives from both ongoing lived experiences and past recovery journeys are incorporated into the study conduct and dissemination of the results. We will also evaluate the feasibility of including an individual with risk factors for an ED or who currently has clinical symptoms of an ED in the study, ensuring that any participation is ethically managed with appropriate safeguards in place to protect the participant well-being. In consultation with our PWLE collaborators, we will explore the possibility of building structured reflective spaces and additional sources of support into the protocol to ensure that participation is safe and meaningful for all involved.

These individuals have been involved from the study protocol stage onwards, contributing to refining the research questions, search strategy, inclusion and exclusion criteria, and outcome prioritization. Moving forward, they will continue to be involved in NMA results interpretation, dissemination efforts, and stakeholder engagement, ensuring that the findings are accessible and meaningful to both academic and non-academic audiences. Their involvement will also include writing a lay summary and co-developing communication strategies to maximize the research’s reach and impact.

Additionally, PWLE collaborators will be asked to comment on the likely efficacy and real-world applicability of the most effective interventions identified in the NMA, providing insights into their feasibility, accessibility, and potential for meaningful impact. We will ask them the following specific questions, especially for the discussion section of the main NMA publication:

- Relevance of Issues: What do you think are the main obstacles to preventing EDs/ delivering prevention interventions?
- Benefits of Prevention: What are/would be the benefits of preventing EDs, from your experience? How important is it that we focus efforts on prevention?
- Intervention Preferences: What would the main features of a good prevention intervention be – what would it focus on and how would it help?
- Barriers: Can you share any real-world experiences or barriers that have affected the success of prevention strategies?
- Suggestions for Improvement: How can existing prevention programs be improved to better meet your needs and those of others at risk for EDs?
- Engagement Strategies: What methods would increase your engagement and adherence to prevention programs? What might make treatment more accessible and acceptable to you?
- Feedback on Findings: Do the preliminary findings and conclusions align with your experiences and expectations? How can they be made more relevant? How can they best be communicated to a diverse audience?
- Effectiveness of Interventions: Based on your lived experience, do you think that the interventions identified as most effective in the NMA would be helpful in real-world settings? What potential challenges or barriers might reduce their effectiveness? How can these interventions be improved to better support people at risk for EDs?
- Safety Concerns: Have you encountered or heard of any instances where participation in preventive or educational events related to ED led to unintended consequences, such as the onset or worsening of disordered eating behaviors? What safeguards would you recommend to ensure that such interventions do not inadvertently contribute to the development of EDs?

These questions help gather valuable qualitative insights to enhance the relevance and applicability of our NMA. Additionally, the project team is committed to inviting and integrating feedback from our PWLE and at-risk status members throughout the research process, in relation to any factors they perceive as being important.

In writing up our findings, we will follow the GRIPP2 (Guidance for Reporting Involvement of Patients and the Public) guidelines [48].

### Dissemination

We intend to publish findings from this NMA in a peer-reviewed scientific journal, along with providing open access to the dataset. Additionally, we will distribute the completed review through electronic channels, print publications, and social media platforms as deemed suitable. Furthermore, we will present and discuss the data at national and international conferences.

We will also present the results of this work in front of policymakers. Coproducing outputs with PWLE and at-risk status members will help make our dissemination efforts more impactful for a wider range of audiences.

## Discussion

EDs present significant challenges in both clinical management and public health efforts. Previous research underscores the importance of prevention strategies in mitigating the onset and progression of EDs. While existing meta-analyses and reviews have provided valuable insights into the effectiveness of various interventions, there remains a need for a comprehensive synthesis of evidence, particularly through an NMA approach.

This study protocol outlines a systematic review and NMA that will contribute to the advancement of ED prevention research and clinical practice in several ways. By addressing methodological limitations of previous reviews and considering data from a wide range of prevention approaches, outcomes, and questionnaires, the NMA will offer a nuanced understanding of intervention efficacy across different prevention levels contributing to a more robust evidence base for ED prevention efforts.

The findings of this study will have significant implications for both research and clinical practice in the field of ED prevention. They will offer valuable guidance for healthcare providers, policymakers, and educators in selecting the most appropriate interventions for specific populations and prevention types. Moreover, the study will identify research gaps in ED prevention, providing insights for future research directions and informing the development of novel prevention strategies.

Our NMA has several strengths. By considering a diverse array of prevention approaches, outcomes, and measurement tools, our NMA ensures a comprehensive assessment of interventions for ED prevention. This breadth of inclusion enhances the applicability and generalizability of our findings, providing valuable insights for clinicians, researchers, and policymakers. While psychological and educational approaches dominate ED prevention research, lifestyle-based interventions, including physical activity, may also help reduce ED risk and prevent ED onset. However, these approaches have remained underrepresented in prior reviews. By incorporating lifestyle interventions, our study assesses their effectiveness and informs future prevention strategies that integrate psychological and lifestyle-based components. This is particularly important as for example increased physical activity can be part of the behavior leading to an ED or maintaining it [19]. The utilization of two independent reviewers for screening studies and data extraction enhances the rigor and reliability of our NMA. This approach minimizes the risk of bias and ensures consistency in the selection and extraction process, thereby strengthening the validity of our results. The careful integration of both PLWE and at-risk status individuals throughout lends strength and novelty to this project. It is particularly innovative to include the perspectives of those who would benefit from access to evidence-based and effective prevention interventions, as well as the insights of individuals who have experienced preventable adverse outcomes during their illness and treatment.

However, our NMA also faces some limitations. While we refer to our work as focusing on prevention, it is important to acknowledge that the majority of studies in the ED field may not align with the standard definition of prevention studies, particularly in terms of preventing the onset of a disorder. This discrepancy may introduce limitations in interpreting the effectiveness of interventions solely in terms of prevention. Due to the nature of available evidence, our NMA may predominantly include RCTs focusing on risk factor reduction rather than pure prevention studies. This limitation underscores the need for caution in extrapolating findings to pure prevention contexts and highlights the complexity of evaluating interventions in the ED prevention field. While indirect comparisons in NMAs respect randomization, it is important to acknowledge that they do not constitute randomized evidence themselves. This criticism raises considerations about the interpretation and generalizability of findings derived from indirect comparisons, emphasizing the need for careful interpretation and contextualization of results. Additionally, the evidence from this NMA of RCTs should also be followed up by and confirmed in real-world evidence studies in patients treated in usual care settings.

Despite these limitations, the study’s findings will have significant implications for ED prevention research and practice, informing research gaps and future research directions, and potentially enhancing the well-being of individuals at risk and reducing the burden of these disorders on individuals and society.

## Electronic supplementary material

Below is the link to the electronic supplementary material.

Supplementary Material 1

Supplementary Material 2

## Data Availability

No datasets were generated or analysed during the current study.

## Abbreviations

AN: Anorexia nervosa
BED: Binge-eating disorder
BMI: Body mass index
BN: Bulimia nervosa
CBT: Cognitive behavioral therapy
CI: Confidence interval
DB: Dissonance-based
DSM: Diagnostic and statistical manual of mental disorders
EAT: Eating attitudes test
ED: Eating disorder
EDDI: Eating disorder diagnostic interview
EDDS: Eating disorder diagnostic scale
EDE: Eating disorder examination interview
EDE-Q: Eating disorder examination questionnaire
EDI: Eating disorder inventory
EOT: End of treatment
f2f: Face-to-face
GRIPP2: Guidance for reporting involvement of patients and the public
HR: Hazard ratio
ICD: International statistical classification of diseases and related health problems
LGBTQ +: Lesbian, gay, bisexual, transgender, queer or questioning, and others
NMA: Network meta-analysis
NNT: Number-needed-to-treat
OR: Odds ratio
PRISMA-P: Preferred reporting items for systematic review and meta-analysis protocols
PWLE: People with lived experience
RCT: Randomized controlled trial
RoB2: Risk-of-bias
RR: Risk ratio
SD: Standard deviation
SE: Standard error
SUCRA: Surface under the cumulative ranking curve
%mBMI: Percentage of median BMI
