# Supplementary material for: Comparative efficacy and acceptability of interventions for universal, selective and indicated prevention of eating disorders: study protocol for a systematic review and network meta-analysis
Source: J Eat Disord. 2025 Apr 25;13:72. doi: 10.1186/s40337-025-01244-8 (PMC12032746; doi:10.1186/s40337-025-01244-8)
Supplement: Supplementary file 1 — Supplementary Material 1 [file 40337_2025_1244_MOESM1_ESM.docx]

**Supplemental Table 1: Codebook for the Categorization of Questionnaires by Outcomes**

| **Outcome** | **Questionnaire** | **Abbreviation** | **Total or Subscale** |
| --- | --- | --- | --- |
| **ED Symptoms/ED Diagnostic Symptoms** | Eating Disorder Diagnostic Interview | EDDI | Total |
|  | Computerized Diagnostic Interview Schedule for Children | C-DISC | Total |
|  | Eating Disorder Diagnostic Scale | EDDS | Total |
|  | Questionnaire for Eating Disorder Diagnosis | Q-EDD | Total |
|  | Eating Disorder Examination-Interview – Diagnostic Items  Eating Disorder Examination-Questionnaire  – Diagnostic Items | EDE-DI (no official abbreviation)  EDE-Q-DI (no official abbreviation) | Subscale  Subscale |
|  | SCOFF (used as continuous measure) | SCOFF | Total |
| **Overall Eating Psychopathology** | EDE  EDE-Q | EDE  EDE-Q | Total  Total |
|  | Eating Attitude Test | EAT | Total |
|  | Eating Disorder Inventory | EDI | Total |
|  | Structured Inventory for Anorexic and Bulimic Eating Disorders | SIAB-S | Total |
|  | EDI – Eating Disorder Risk Composite (Subscales Drive for Thinness + Bulimia + Body Dissatisfaction) | EDI-EDRC | Total |
| **Body Dissatisfaction** | Body Shape Questionnaire | BSQ | Total |
|  | Satisfaction and Dissatisfaction with Body Parts Scale | SDBPS (no official abbreviation) | Total |
|  | Body Image Avoidance Questionnaire | BIAQ | Total |
|  | Multidimensional Body-Self Relations Questionnaire  MBSRQ – Appearance Evaluation  MBSRQ – Appearance Orientation  MBSRQ – Body Areas Satisfaction Scale | MBSRQ  MBSRQ-AE  MBSRQ-AO  MBSRQ-BASS | Total  Subscale  Subscale  Subscale |
|  | Beliefs About Appearance Scale | BAAS | Total |
|  | EDI – Body Dissatisfaction | EDI-BD | Subscale |
| **Body Satisfaction / Body Appreciation** | Body Esteem Scale (for Adolescents and Adults)  BESAA – Appearance  BESAA – Weight  BESAA – Attribution | BES /BESAA  BESAA-AP  BESAA-W  BESAA-A (no official abbreviation) | Total  Subscale  Subscale  Subscale |
|  | Body Appreciation Scale | BAS | Total |
|  | Body Parts Satisfaction Scale | BPSS | Total |
|  | Satisfaction with Body Parts Scale | SBPS | Total |
| **Weight Concerns** | Weight Concerns Scale | WCS | Total |
|  | Killen Measure of Weight Concern | KWC | Total |
|  | EDE – Weight Concern  EDE-Q – Weight Concern | EDE-WC  EDE-Q-WC | Subscale  Subscale |
|  | BES – Weight Concern | BES-WC | Subscale |
| **Shape Concerns** | EDE – Shape Concern  EDE-Q – Shape Concern | EDE-SC  EDE-Q-SC | Subscale  Subscale |
| **Weight AND Shape Concerns** | Weight and Shape Concerns Scale | WSC | Total |
|  | EDE – Weight Concern and Shape Concern combined  EDE-Q – Weight Concern and Shape Concern combined | EDE-WSC  EDE-Q-WSC | Subscale  Subscale |
| **Drive for Thinness** | EDI – Drive for Thinness | EDI-DT | Subscale |
| **Thin-ideal internalization** | Ideal-Body Stereotype Scale | IBSS | Total |
|  | Thin Ideal Internalization Scale | TIIS (no official abbreviation) | Total |
|  | SATAQ-4 – Internalization - Thin/low body fat  SATAQ-4 – Internalization - Muscular/Athlete | SATAQ-4-I-T  SATAQ-4-I-M | Subscale  Subscale |
| **Dieting** | Dutch Restrained Eating Scale | DRES | Total |
|  | Cognitive Behavioral Dieting Scale | CBDS | Total |
|  | EDE-Q – Restraint | EDE-Q-R | Subscale |
|  | Dutch Eating Behavior Questionnaire – Restraint | DEBQ-R | Subscale |
|  | EAT – Dieting | EAT-D | Subscale |
| **Eating Concern** | EDE – Eating Concern  EDE-Q – Eating Concern | EDE-EC  EDE-Q-EC | Subscale  Subscale |
|  | ChEAT – Food preoccupation | ChEAT-FP | Subscale |
| **Body Mass Index** | Body Mass Index | BMI | Total |
| **Bulimia/Bulimic Symptoms (mixed: cognitive and behavioral items)** | Bulimia Test | BULIT | Total |
|  | Binge Eating Scale | BES | Total |
|  | EDI – Bulimia | EDI-B | Subscale |
|  | EAT – Bulimia and food preoccupation  ChEAT – Restricting & Purging | EAT-B  ChEAT-RP | Subscale  Subscale |
| **ED Behavioral Symptoms (single items)** (bingeing,  purging,  laxatives, exercise,  diet pill use) | EDE  EDE-Q | EDE  EDE-Q | Single items |
|  | Short Evaluation of Eating Disorders | SEED | Single items |
|  | Eating Disorders Diagnostic Scale | EDDS | Single items |
|  | SCID Symptom Checklist | SCID | Single items |
|  | Extreme Weight Loss Behaviours Scale | EWLB | Total |
| **Media internalization** | Sociocultural attitudes towards appearance questionnaire - Internalization  SATAQ-3 – Internalization - General | SATAQ-IN  SATAQ-3-IN-G | Subscale  Subscale |
| **Depressive symptoms** | Beck Depression Inventory | BDI | Total |
|  | Children’s Depression Inventory | CDI | Total |
|  | Depression, Anxiety, Stress Scale - Short Form – Depression | DASS-21-D | Subscale |
|  | Patient Health Questionnaire-9 | PHQ-9 | Total |
|  | Center for Epidemiologic Studies – Depression | CES-D | Total |
|  | Allgemeine Depressionsskala (German version of CES-D) | ADS | Total |
| **Negative affect** | Positive and Negative Affect Schedule  PANAS – Negative affect  PANAS – Positive affect | PANAS  PANAS-NA  PANAS-PA | Total  Subscale  Subscale |
|  | Depression, Anxiety, Stress Scale | DASS | Total |
|  | Kessler 10-item distress scale | K-10 | Total |
| **Anxiety** | Generalized Anxiety Disorder Scale | GAD-7 | Total |
|  | Multidimensional Anxiety Scale for Children | MASC | Total |
|  | State Trait Anxiety Inventory | STAI | Total |
|  | Patient-Reported Outcomes Measurement Information System anxiety short-form version 1.0 questionnaire | PROMIS-A (no official abbreviation) | Total |
|  | DASS - Short Form – Anxiety | DASS-21-A | Subscale |
| **Self-esteem** | Rosenberg Self-Esteem Scale | RSES | Total |
|  | Self-Esteem Inventory for Children | SEI | Total |
|  | EDI-2 – Ineffectiveness Subscale | EDI-I | Subscale |
| **Self-compassion** | Self-Compassion Scale | SCS | Total |
