# Supplementary material for: Comparative efficacy and acceptability of interventions for universal, selective and indicated prevention of eating disorders: study protocol for a systematic review and network meta-analysis
Source: J Eat Disord. 2025 Apr 25;13:72. doi: 10.1186/s40337-025-01244-8 (PMC12032746; doi:10.1186/s40337-025-01244-8)
Supplement: Supplementary file 2 — Supplementary Material 2 [file 40337_2025_1244_MOESM2_ESM.docx]

**Supplemental Material 2**

**Search strategy**

[Ovid MEDLINE(R) ALL <1946 to September 03, 2024>](https://login.proxy.bib.uottawa.ca/login?url=http://ovidsp.ovid.com/ovidweb.cgi?T=JS&NEWS=N&PAGE=main&SHAREDSEARCHID=41IuMCj5dRViwANIV6JT43Juv5UEvl2N2D130aCkWcsHQepddRcQIvVT81aTqJj2G)

1 "feeding and eating disorders"/ or anorexia nervosa/ or binge-eating disorder/ or bulimia nervosa/ 35364

2 (eating disorder* or disorder* eating or anorexi* or bulimi* or binge eating).tw,kf. 65999

3 Body Image/ 20389

4 body image.tw. 14343

5 1 or 2 or 3 or 4 93275

6 randomized controlled trial.pt. 620839

7 (random* or placeb*).tw. 1636841

8 trial.ti. 317331

9 control group*.tw,kf. 611705

10 controls.tw,kf. 1027410

11 or/6-10 3096698

12 5 and 11 14633

13 pc.fs. 1506289

14 prevent*.tw,kf. 1901492

15 intervent*.tw,kf. 1493112

16 13 or 14 or 15 4096105

17 12 and 16 3708

18 exp animals/ not humans/ 5254851

19 17 not 18 3545

20 exp neoplasms/ or (cancer or neoplasm* or carcinoma).ti. 4316476

21 "feeding and eating disorders"/ or anorexia nervosa/ or binge-eating disorder/ or bulimia nervosa/ 35364

22 20 not 21 4315643

23 19 not 22 3161

24 limit 23 to dt=20240101-20240904 201
